# SO_2_ Capture Using Porous Organic Cages

**DOI:** 10.1002/anie.202104555

**Published:** 2021-06-10

**Authors:** Eva Martínez‐Ahumada, Donglin He, Victoria Berryman, Alfredo López‐Olvera, Magali Hernandez, Vojtech Jancik, Vladimir Martis, Marco A. Vera, Enrique Lima, Douglas J. Parker, Andrew I. Cooper, Ilich A. Ibarra, Ming Liu

**Affiliations:** ^1^ Laboratorio de Fisicoquímica y Reactividad de Superficies (LaFReS) Instituto de Investigaciones en Materiales Universidad Nacional Autónoma de México Circuito Exterior s/n, CU Coyoacán 04510 Ciudad de México Mexico; ^2^ Department of Chemistry, Materials Innovation Factory Leverhulme Centre for Functional Materials Design University of Liverpool Liverpool L69 7ZD UK; ^3^ Centro Conjunto de Investigación en Química Sustentable UAEM-UNAM Carretera Toluca-Atlacomulco km 14.5 C.P.50200 Toluca Estado de México Mexico; ^4^ Universidad Nacional Autónoma de México Instituto de Química Circuito Exterior s/n, CU Coyoacán 04510 Ciudad de México Mexico; ^5^ Surface Measurement Systems Unit 5, Wharfside, Rosemont Road London HA0 4PE UK; ^6^ Universidad Autónoma Metropolitana-Iztapalapa San Rafael Atlixco 186, Col. Vicentina Iztapalapa C. P. 09340 Ciudad de México Mexico

**Keywords:** adsorption, chemical stability, porous organic cages, SO_2_

## Abstract

We report the first experimental investigation of porous organic cages (POCs) for the demanding challenge of SO_2_ capture. Three structurally related N‐containing cage molecular materials were studied. An imine‐functionalized POC (CC3) showed modest and reversible SO_2_ capture, while a secondary‐amine POC (RCC3) exhibited high but irreversible SO_2_ capture. A tertiary amine POC (6FT‐RCC3) demonstrated very high SO_2_ capture (13.78 mmol g^−1^; 16.4 SO_2_ molecules per cage) combined with excellent reversibility for at least 50 adsorption–desorption cycles. The adsorption behavior was investigated by FTIR spectroscopy, ^13^C CP‐MAS NMR experiments, and computational calculations.

## Graphical abstract

Porous organic cages (POCs) were investigated for the first time for the capture of SO_2_. A tertiary amine cage (6FT‐RCC3) demonstrated remarkably high SO_2_ capture that was perfectly reversible for at least 50 adsorption–desorption cycles.

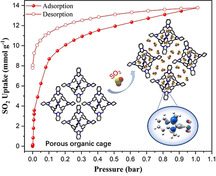

## Introduction

Modern society faces critical challenges related to controlling the release of toxic pollutants into the atmosphere. Air pollution reduction is a fundamental part of strategies to tackle climate change.^[1]^ Air pollution produces a large variety of health problems (e.g., morbidity and premature death)^[2]^ and it also accounts for decreases in biodiversity, water acidification and crop damage.^[3]^ Sulphur dioxide (SO_2_), is a colourless, irritating and non‐flammable gas with a sharp odour, which can be absorbed through the respiratory system or by dermal contact.^[4]^ SO_2_ is highly toxic to humans, and exposures over 100 ppm can be deadly.^[5]^ The frightening increase in SO_2_ emissions by anthropogenic activities such as fossil fuel combustion^[6]^ creates an urgent for immediate environmental remediation action. In fact, the World Health Organization (WHO) has classified SO_2_ as one of the most hazardous air pollutants with catastrophic health effects, correlated primarily to severe modifications of the respiratory system (e.g., broncho‐constriction in lung function).^[7]^ For example, if a healthy person is exposed to a SO_2_ concentration of 1.5 ppm for just a few minutes, it can produce a temporary inability to breathe.^[8]^ At slightly higher concentrations, SO_2_ can cause laryngitis, chronic bronchitis and severe infections of the respiratory tract.^[9]^ Air quality guidelines advise the maximum values for human exposure to SO_2_ to be 500 μg m^−3^ (175 ppb) over 10 min and 20 μg m^−3^ (8 ppb) for daily averages.^[10]^ To comply with these standards, significant quantities of SO_2_ must be removed from our environment to ensure both human health protection and environment preservation, particularly in urban areas.

One of the first techniques to remove SO_2_, spiral‐tile packed tower, was developed in 1933.^[11]^ Currently, the most common strategies for SO_2_ removal from industrial combustion units are scrubbers. Typically, electricity power plants employ desulphuration methods based on aqueous alkaline solutions and/or wet‐sulphuric‐acid processes.^[12]^ However, these methods create huge quantities of wastewater, corrosion of pipelines, substantial cost of use and recovery, and leave traces of SO_2_ (approximately 400 ppm^[13]^), posing a foremost health risk according to the WHO.^[14]^ As a result of these disadvantages, we need to explore more efficient and effective technologies for the capture of SO_2_, and solid state materials show potential to overcome many of these pitfalls. For example, the removal of SO_2_ has been investigated with zeolites,^[15]^ but requirement for high temperature (450 °C) and helium flow during the process are drawbacks.^[16]^ Other examples of solid materials that have been investigated for SO_2_ capture are metal oxides,^[17]^ however, strong SO_2_ interactions (chemisorption) leads to an irreversible structure transformation,^[18]^ which again impedes their regeneration.

Thus, the development of new materials capable of adsorbing, preferably physisorbing, high quantities of SO_2_ is being increasingly investigated.^[19]^ For example, hybrid porous materials such as Metal‐Organic Frameworks (MOFs), have been investigated for the capture of SO_2_ with some promising results from limited numbers of chemically‐stable MOFs to SO_2_ (e.g., MFM‐300(Al),^[20a]^ MFM‐300(In),^[20b]^ MFM‐300(Sc),^[20c]^ and MIL‐101(Cr)‐4F(1 %).^[21]^ However, the potentially high cost of production for the organic components, combined with (sometimes) challenging scalability makes deployment of these materials difficult. Perhaps the biggest hurdle is the poor chemical stability of many MOFs (and indeed other materials) to SO_2_,^[22]^ which is an aggressively corrosive gas. This poses questions for the economics of capturing SO_2_ using MOFs on industrial scales.

Porous organic cages (POCs), first reported in 2009, are an emerging subclass of porous materials that are permanently microporous in the solid state.^[23]^ Unlike porous frameworks, such as zeolites, MOFs or covalent‐organic‐frameworks (COFs), the discrete cage molecules are solution processable, and can be used as tectons in the modular construction of highly porous crystalline materials.^[24]^ POCs have been explored in various applications related to gas storage and separation. For example, it has been demonstrated that POCs are promising adsorbents for greenhouse gases (SF_6_),^[25]^ rare gases, and radioisotope pollutants.^[26]^ Of particular relevance here, relatively simple chemical^[27]^ or crystal engineering modification^[28]^ can lead to POC materials that are exceptionally stable under both acidic and basic conditions. POCs can be easily processed into composite membranes,^[29]^ thin films,^[30]^ and stationary phases for chromatography separation.^[31]^ In the past few years, significant progress has also been made on scaling up specific POC materials, through processes including in batch,^[32]^ flow syntheses,^[33]^ microwave‐assisted synthesis^[34]^ and twin screw extrusion.^[35]^

## Results and Discussion

**Figure 1:**
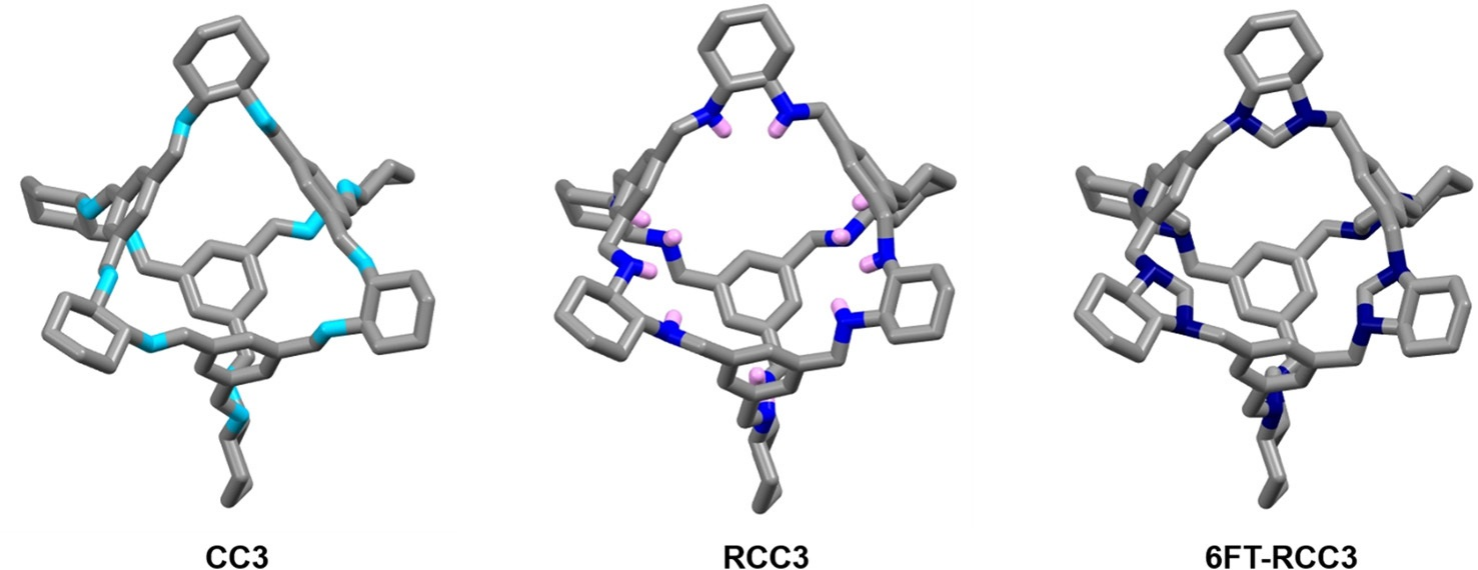
Crystal structures (top) and chemical structures (bottom) for porous cage CC3 (a), RCC3 (b), and FT‐RCC3 (c). Carbon and nitrogen atoms are shown in grey and blue, respectively. Hydrogen atoms are omitted for clarity, except in at the secondary amine group of RCC3, where hydrogen is shown in whitepink.^[28]^

The key to adsorbing large amounts of SO_2_ under practical relevant conditions is the careful selection of functional groups that have a high affinity for this acidic gas, as demonstrated in other porous materials that contain ‐OH or ‐NH_2_ groups.^[36]^ Furthermore, it has been demonstrated that the adsorption of SO_2_ is preferred on surfaces with N‐containing functional groups^[37]^ and its reversibility strongly depends on the basicity of the N species in the adsorbent.^[38]^ In fact, most of the materials used in industrial desulphurization technologies are amine solutions.^[39]^ Taking that into account, we decided to investigate a series of molecular cages as SO_2_ adsorbents with three different N‐containing functional groups: imine, CC3; secondary amine, RCC3; and tertiary amine functionalization, 6FT‐RCC3, (Figure 1).[^23^, ^27^, ^41^] As can be seen in Figure 1, it is possible to obtain high densities of nitrogen atoms (shown in blue) in these cage materials. It is worth noting that, the three cages are isostructural in crystalline form, with almost identical size, shape and packing mode in solid state (Figure 1).

**Figure 2:**
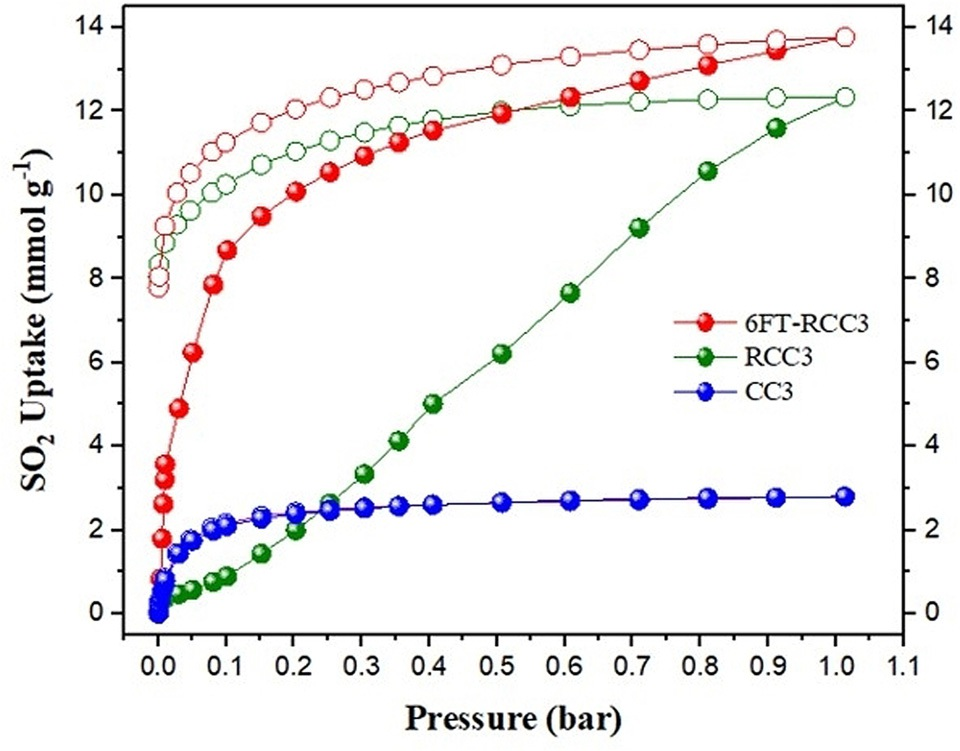
SO_2_ adsorption isotherms of CC3 (blue isotherm), RCC3 (green isotherm) and 6FT‐RCC3 (red isotherm) at 298 K and 1 bar. Closed symbols (adsorption isotherm), open symbols (desorption isotherm).

The first molecular cage studied was CC3, this structure contains 12 imine groups per molecular cage (Figure 1 a). CC3 showed a SO_2_ uptake of 2.78 mmol g^−1^ at 1 bar and 298 K; the isotherm is shown in Figure 2. The SO_2_ adsorption capacity agrees (approximately) with the grand canonical Monte Carlo simulations carried out by W. Li and J. Zhang, where the adsorption of different acidic gases in CC3 was studied computationally.^[41]^ The simulated isotherm for SO_2_ shows an approximate total adsorption of 3.6 mmol g^−1^ at 1 bar and 275 K, and an atypical type I shape, associated to Coulomb interaction for SO_2_⋅⋅⋅SO_2_ complexes.^[41]^ In our case, the experimental adsorption follows a characteristic type‐I isotherm without hysteresis that it is associated with the reversibility of the process; that is, physisorption of the gas molecule inside the cages. Somewhat surprisingly given its imine bonding, retention of crystallinity was observed by PXRD after the exposure to SO_2_ (ESI Figures S1a). We also carried out an SO_2_ adsorption experiment at 308 K to calculate the heat of adsorption (see Table 1 and ESI Figure S2a). The resultant *Q*st was equal to 38.46 kJ mol^−1^ which is characteristic for a physisorption rather than a chemisorption processes.^[42]^ The SO_2_ adsorption capacity for CC3 does not compete well with various stable MOFs. The linear uptake portion of the isotherm also implies poor adsorption kinetics and lack of equilibration.

**Table 1:** SO_2_ adsorption quantities for each cage material at 298 K, 1 bar.

Sample	SO_2_ Uptake Capacity [mmol g^−1^]	SO_2_ Packing Density [g cm^−3^]	Heat of Adsorption [kJ mol^−1^]
CC3^[a]^	2.78	0.18*	38.46
RCC3^[a]^	12.34	–	82.78
6FT‐RCC3^[a]^	13.78	0.91*	43.03
MFM‐300(In)^[20b]^	8.28	1.27	34.5
MIL‐101(Cr)‐4F(1 %)^[21]^	18.4	0.99	54.3
MIL‐125(Ti)‐NH_2_[^36a^, ^36b^]	10.8 (3.0)^[c]^	1.06	53
MOF‐177^[36a]^	25.7^[d]^	1.09	–
[Zn_2_(L_1_)_2_(bipy)]^[36d]^	10.9^[d]^	11.84	–
MFM‐170^[43]^	17.5	1.27	35.4
SIFSIX‐3‐Zn^[44a]^	2.1 (1.68)^[b]^	–	45.2
SIFSIX‐3‐Ni^[44a]^	2.74 (2.43)^[b]^	0.86	43.2
SIFSIX‐1‐Cu^[44a]^	11.01 (3.43)^[b]^	1.64	36.1
SIFSIX‐2‐Cu‐i^[44a]^	6.9 (4.16)^[b]^	1.43	38.1
P(Ph‐4MVIm‐Br)^[44b]^	8.12 (2.43)^[b]^	4.34	76–65.2^[e]^
PI‐COF‐m^[51c]^	6.5	–	–

[a] 12 N atoms per porous cage. [b] 12 BDC‐NH_2_ linkers per unit cell. [c] at low pressure of 0.01 bar. [d] measured at 293 K. [e] DFT calculations. * Packing density was calculated considering a previously reported pore volume of 0.973 cm^3^ g^−1^ in both cases.^[24]^

The next material analyzed was a secondary amine‐cage RCC3 (Figure 1 b). RCC3 showed a much higher SO_2_ uptake capacity of 12.34 mmol g^−1^ at 298 K up to 1 bar, comparable to the current best performing MOF materials for this application, such as MFM‐601,^[43a]^ SIFSIX‐1‐Cu,^[44a]^ [Zn_2_(L_1_)_2_(bipy)],^[36c]^ and MFM‐202a^[43b]^ (12.3, 11.0, 10.9, and 10.2 mmol g^−1^, respectively). The SO_2_ isotherm of RCC3 exhibited an open loop hysteresis (Figure 2), indicating limited reversibility with this secondary amine material.

The highest SO_2_ capture was achieved with 6FT‐RCC3, reaching a maximum uptake of 13.68 mmol g^−1^ (Figure 2), this uptake is only behind the reported benchmark MOFs such as MOF‐177,^[36a]^ MIL‐101(Cr) 4F(1 %),^[21]^ or MFM‐170^[43c]^ (25.7, 18.4 and 17.5 mmol g^−1^, respectively). It is worth noting that the BET surface area of abovementioned three MOFs all exceeds 2000 m^2^ g^−1^, while for 6FT‐RCC3 is 396 m^2^ g^−1^. A comparison of SO_2_ uptakes and the BET surface area of some representative MOF materials is provided in Figure S3 which highlights the highly competitive SO_2_ capture performance of 6FT‐RCC3 cage, considering its modest surface area. Interestingly, 6FT‐RCC3 shows a significant SO_2_ capacity at low SO_2_ pressures: at 0.1 bar 6FT‐RCC3 captures 8.67 mmol g^−1^ of SO_2_. This uptake is slightly, higher than the value of 8.28 mmol g^−1^ at 1 bar and 298 K observed for MFM‐300(In),^[20b]^ a MOF material with a superior surface area (approximately 1100 m^2^ g^−1^). When comparing the SO_2_ uptake by 6FT‐RCC3 at low partial pressures (e.g., 0.01 bar, 0.15 bar and 0.5 bar) the SO_2_ capacities were 3.57 mol g^−1^; 9.48 mmol g^−1^ and 11.94 mmol g^−1^ which outperforms several MOF materials such as: MFM‐170 (≈6.5 mmol g^−1^ at 0.1 bar),^[43]^ SIFSIX‐3‐Ni (2.43 mmol g^−1^ at 0.01 bar),^[44a]^ MOF‐177 (0.3 mmol g^−1^ at 0.01 bar, 1.0 mmol g^−1^ at 0.1 bar),^[36a]^ MIL‐125(Ti)‐NH_2_ (3.0 mmol g^−1^ at 0.01 bar, 7.9 mmol g^−1^ at 0.1 bar);^[36a]^ as well as various polymers/COF materials, such as CTF‐CSU41 (6.7 mmol g^−1^ at 0.15 bar) and CTF‐CSU38 (4.4 mmol g^−1^ of at 0.15 bar);^[45]^ ionic microporous polymer P(Ph‐4MVIm‐Br) (2.43 mmol g^−1^ at 0.01 bar and 4.14 mmol g^−1^ at 0.1 bar);^[44b]^ and it is comparable to MOF SIFSIX‐1‐Cu (3.43 SO_2_ mmol g^−1^ at 0.01 bar) (see Figure S4).^[44a]^ This remarkable uptake at low SO_2_ partial pressures indicates the possibility of using solid 6FT‐RCC3 for trace SO_2_ capture.

6FT‐RCC3 shows a type‐I isotherm with a moderate degree of hysteresis (Figure 2). Because of the molecular flexibility of both 6FT‐RCC3 and RCC3, open loop hysteresis occurs due to swelling effects as observed previously for similar materials.^[46]^ The heat of adsorption at zero coverage for each sample is shown in Table 1. The high *Q*_st_ value for RCC3 indicates a strong bond between the functional R_2_N‐H amine group in RCC_3_ and SO_2_ which suggests an almost irreversible chemisorption process. The experimental heat of adsorption values obtained for RCC3 and 6FT‐RCC3 (82.78 and 43.03 kJ mol^−1^, respective), are in good agreement with different N‐based adsorbents such as diamines,^[47]^ Merrifield resins,^[48]^ ionic liquids,^[49]^ and hybrid solvents.^[50]^ The preferential adsorption binding site in all cases is the N atom, forming a charge‐transfer complex with SO_2_ (N→SO_2_).[^50^, ^51^]

**Figure 3:**
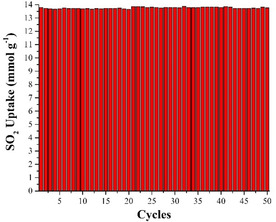
Fifty adsorption‐desorption cycles for SO_2_ in 6FT‐RCC3 at 298 K. SO_2_ was fully desorbed under dynamic vacuum at 353 K between cycles. No loss of uptake capacity was observed.

To corroborate the reversibility of the process, we carried out SO_2_ cyclability experiments on these materials. In the case of RCC3, we only managed to obtain three partial adsorption‐desorption cycles of SO_2_ (activating under dynamic vacuum with and without heating, see ESI Figure S5). In fact, when RCC3 and 6FT‐RCC3 were only activated under vacuum at room temperature, we observed a decrease in SO_2_ uptake after the first cycle in both cases. However, re‐activation at 80 °C under vacuum for 6FT‐RCC3 shows full retention of the adsorption capacity of SO_2_ after 50 adsorption‐desorption cycles at 298 K (Figure 3 for 6FT‐RCC3 and Figure S6 for CC3). These results show that the SO_2_ affinity follows the trend: tertiary amine > secondary amine > imine, in good agreement with the basicities of the cage.^[48]^ The corroborates that the inclusion of tertiary amines in these porous materials allows higher SO_2_ uptake. It is also worth noting that structure of 6FT‐RCC3 is intact after 50 SO_2_ adsorption‐desorption cycles as confirmed by solution NMR experiments (ESI, Figure S10 and S11).

PXRD analyses of RCC3 and 6FT‐RCC3 after SO_2_ exposure confirm a significant loss in crystallinity. We believe that for RCC3, the high heat of adsorption for SO_2_ and the lack of recyclability of the material indicates a chemical transformation of the structure (chemical bonding, N→SO_2_), which may collapse the structure. Conversely, the loss of crystallinity for 6FT‐RCC3 can be attributed to the flexibility of the material and the relatively strong interaction with SO_2_ molecules, as demonstrated by the heat of adsorption and high sorption cyclability (see Figure 3). We speculate that SO_2_ molecules might be accommodated into the pore structure of 6FT‐RCC3 as result of the high affinity, even after all the voids in the structure of the crystalline phase have been filled, and eventually disrupt the regular packing. We note that amorphous POCs of this type can be more porous than their crystalline analogues in some cases,^[32]^ and hence loss of crystallinity is not necessarily a disadvantage‐a key distinguishing feature between these inherently molecular solids and frameworks such as MOFs and COFs, which typically lose their porosity when then become amorphous. This may be particularly beneficial for separations that involve strongly interacting and chemically reactive guests such as SO_2_.

**Figure 4:**
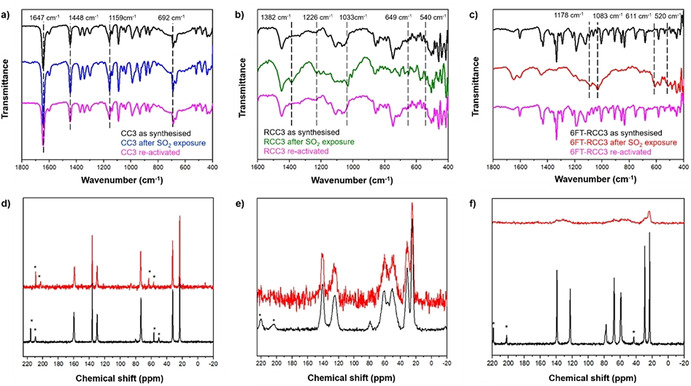
a–c) FT‐IR spectra of as‐synthesised, SO_2_‐loaded, and regenerated a) CC3, b) RCC3, and c) 6FT‐RCC3, split into 1800–400 cm^−1^ wavelength region. Dashed lines in (a) shows the strongest vibrational frequencies assigned to C=N, CH_2_, C−N, and C−H stretching modes. Dashed lines in (b) and (c) are a visual guide to the new bands observed after SO_2_ exposure. d–f) ^13^C CP MAS NMR spectra of as‐synthesised (black line) and SO_2_‐loaded (red line) of d) CC3, e) RCC3, and f) 6FT‐RCC3 porous organic cages. * Indicates spinning side bands (6 kHz).

FTIR spectroscopy experiments were performed on the as‐synthesised, after SO_2_ exposure, and fully re‐activated materials to corroborate the preferential binding sites of the SO_2_ molecule in the cages (see ESI Figure S7). The fundamental vibrational frequencies of the SO_2_ molecule are the symmetric stretch (ν_1_), asymmetric stretch (ν_2_), and bending motion (ν_3_) located at 1153 cm^−1^, 1368 cm^−1^, and 508 cm^−1^, respectively.^[52]^ The interaction of SO_2_ and amine‐based materials can be often be defined, as the formation of a charge‐transfer complex, from the N: lone pair of electrons to the antibonding SO_2_ orbital (N→SO_2_). This interaction causes the appearance of new SO_2_ vibrational bands, as reported for several amines where the formation of charge‐transfer is verified.[^50^, ^51^, ^53^] As shown in Figure 4 a, the strongest vibrational frequencies are assigned to C=N, CH_2_, C−N, and C−H stretching modes at 1654 cm^−1^, 1448 cm^−1^, 1160 cm^−1^ and 690 cm^−1^, respectively.^[54]^ The CC3 spectra before and after SO_2_ adsorption showed no changes, and these results are in good agreement with the adsorption isotherm (vide supra), demonstrating the weak SO_2_ interaction with the CC3 structure. By contrast, the FTIR spectrum for the RCC3 cage shows new bands after SO_2_ adsorption (Figure 4 b, green line). These bands at 1382 cm^−1^ and 649 cm^−1^ are in the range reported for asymmetric stretching and bending for SO_2_ gas,^[52]^ while the vibrational frequencies at 1226 cm^−1^, 1033 cm^−1^ and 540 cm^−1^ are associated to the N‐S interaction and have been reported for NH_3_‐SO_2_.[^55^, ^56^] These results suggest that the adsorption of SO_2_ occurs mainly at the amino groups, while some free SO_2_ interacts as a dimer. Additionally, the molecular cage with tertiary amine functionalisation, 6FT‐RCC3, showed four vibrational frequencies at 1178 cm^−1^, 1083 cm^−1^ 611 cm^−1^ and 520 cm^−1^, which are also related to the formation of an N→SO_2_ complex, see Figure 4 c.

**Figure 5:**
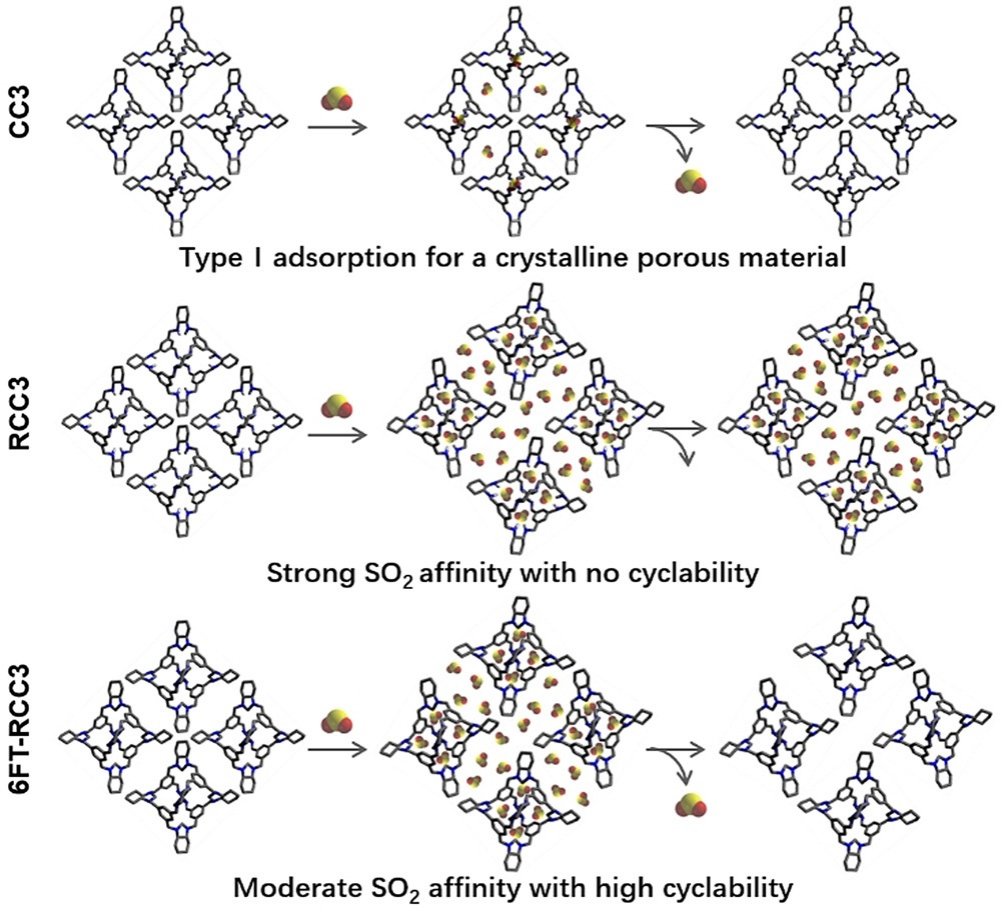
Three types of SO_2_ adsorption behaviours of porous organic cages.

^13^C CP MAS NMR experiments (Figure 4 bottom) showed a good correlation with the FT‐IR for the solid materials. Figure 5 a shows a similar spectrum for the CC3 sample both before and after the SO_2_ adsorption. Both spectra show narrow NMR signals due to aliphatic carbons at 22.8, 32.4 and 73.5 ppm. Three further peaks assigned to aromatic carbons are observed at 130.0, 136.8 and 159.6 ppm. These resonances are similar in breadth and position before and after SO_2_ adsorption, in line with a weak interaction between SO_2_ and CC3. The spectra of RCC3 (Figure 4 b) are composed by peaks assigned to aliphatic carbons at 24.8, 31.1, 50.6 and 61.9 ppm, and by peaks due to aromatic carbons at 125.6 and 140.5 ppm. In contrast with CC3, the NMR peaks corresponding to RCC3 are broad, in keeping with a more flexible molecular solid structure that has less long‐range order. After the SO_2_ adsorption, the mobility and chemical environment of carbons, from the primary units, are significantly modified corroborating a strong interaction with the SO_2_ molecule. However, the isotropic signals are unmodified suggesting that the structure, at least the primary units of this POC, are unchanged. Finally, the spectra corresponding to the 6FT‐RCC3 sample (Figure 4 c) show NMR signals of aliphatic carbons at 23.2, 29.4, 58.9, 67.7 and 78.1 ppm and peaks due to aromatic carbons at 122.6 and 139.1 ppm. The peaks before the SO_2_ adsorption are narrow suggesting an ordered crystalline solid but after the SO_2_ adsorption the peaks became very broad. Signals are observed but the peaks are not sharp or strong enough resolved. It seems that the SO_2_ interaction is strong in this material and that the number of molecules inside the pores significantly modifies the structure and the relaxation of NMR signals.

To better understand of the SO_2_ adsorption mechanism, we carried out theoretical calculations using density functional theory methods and employing the Gaussian 16 software package.^[57]^ Calculations used the PBE^[58]^ density functional approximation with Ahlrich's def2‐TZVP basis set of a polarized triple‐ζ quality.^[59]^ Dispersion was considered with Grimme's D3 dispersion corrections in conjunction with the Becke‐Johnson damping parameters.^[60]^ Geometries were optimized and vibrational frequencies computed to confirm structures were minima on the potential energy surface. All SO_2_ binding energies are reported with zero‐point vibrational energy corrections.

The SO_2_ binding energies were calculated as the difference between the unbound POC moiety and SO_2_ from that of the complex. The structures of the moieties, SO_2_ binding energies, and shortest N‐S distances are shown in Table 2. The SO_2_ binding energies follow the trend of the experimental heats of adsorption, increasing from CC3 (49.7 kJ mol^−1^) to 6FT‐RCC3 (68.6 kJ mol^−1^) to RCC3 (86.4 kJ mol^−1^). These binding energies are greater than for imidazole (39.1 kJ mol^−1^), and a range of imidazole derivatives reported by Shannon et al.^[61]^ The results are again consistent with the notion that binding strength increases with the degree of substitution, since the electron‐donating alkyl groups enhance the nucleophilic character of the N atoms resulting in a greater SO_2_ affinity.

**Table 2:** Structure of the POC moieties, the corresponding SO_2_ binding energies, and shortest N‐S distance to the SO_2_ molecule for each system. All binding is exergonic.

	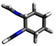	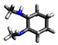	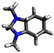
Parent POC	CC3	RCC3	6FT‐RCC3
BE [kJ mol^−1^]	49.7	86.4	68.6
*r*(N‐S) [Å]	2.430	2.390	2.415

Binding was investigated beyond one SO_2_ molecule for the 6FT‐RCC3 moiety and shows that 2 SO_2_ molecules bind per moiety with a negligible change to the binding energy, and (Table 3). This result supports the experimental result of 1:1 binding of SO_2_ to N atoms in the structure.

**Table 3:** Binding energies per SO_2_ molecule to the 6FT‐RCC3 derived moiety, and shortest N‐S distances for each SO_2_ molecule in the system.

No. of SO_2_	BE per SO_2_ [kJ mol^−1^]	R(N‐S) [Å]		
		Min.	Max.	Average
1	68.6	–	–	2.415
2	65.0	2.389	2.395	2.392
3	54.7	2.331	4.770	3.166
4	49.8	2.330	4.854	3.567

Based on the combined experimental and computational results, these three structurally related cages have quite distinct SO_2_ adsorption behaviors, which result from their different functional groups as well as their packing modes in the solid state (Figure 5). CC3 adsorbs SO_2_ molecules like a typical crystalline physisorptive porous solid, where SO_2_ molecules are accommodated in its pore structure without significant adsorbate‐adsorbent interactions. The pore structure of CC3 is unchanged during the SO_2_ adsorption‐desorption cycles.

The amine groups of RCC3 provide very strong binding sites for SO_2_ molecules drawing more gas molecules into the rather flexible pore structure of RCC3. However, those SO_2_ molecules are then hard to desorb from RCC3 structure, because of the high bind affinity between SO_2_ and RCC3 (82.78 kJ mol^−1^). By contrast, the imidazolidine rings on 6FT‐RCC3 seem have on ideal affinity for SO_2_ at 43.03 kJ mol^−1^: they can attract a large amount of SO_2_ into the pore structure, but the adsorbed gas can still be easily removed under dynamic vacuum at 80 °C.

## Conclusion

In summary, we present the first experimental study of porous organic cages for gaseous SO_2_ capture. Three structurally related cage materials were studied, differing only in their functional groups (imines, CC3; secondary amines, RCC3; tertiary amines, 6FT‐RCC3). The three cages have distinct SO_2_ adsorption behaviors, which stems from their very different SO_2_‐adsorbent affinities, as confirmed by adsorption isotherms, FTIR spectroscopy and ^13^C CP MAS NMR experiments. 6FT‐RCC3 showed a remarkable SO_2_ uptake at 13.78 mmol g^−1^ (1 bar, room temperature), rivaling the best performing MOF materials and polymers for this application, as well as showing exceptional stability and cyclability. In particular, high uptake at low partial pressures indicates the potential of 6FT‐RCC3 for trace SO_2_ capture.

The most prevalent technology for SO_2_ today is alkaline scrubbers, but as discussed above, these have numerous disadvantages. Effectively, we have removed the need for an aqueous solvent here by developing a porous organic base. We believe that the modest surface area of these materials is an advantage because their relatively high density means that the volumetric SO_2_ storage capacity is very high. For example, at 298 K/1 bar, 6FT‐CC3 adsorbs 13.78 mmol g^−1^ SO_2_, which equates to 16.4 SO_2_ molecules per cage, on average; that is, one per amine group plus 4.4 others. Since these cages pack a large number of amine groups into a small, compact volume (Figure 1), this equates to an exceptional SO_2_ storage density, in principle allowing for reductions in scale of adsorbers based on these materials. 6FT‐CC3 shows excellent cyclability over at least 50 sorption/desorption cycles (Figure 3) and, unlike many MOFs and other frameworks, loss of crystallinity does not necessarily equate to reduction in porosity for these cage materials.^[32]^ Coupled with good processibility options and, recently, proven synthetic scalability,^[62]^ we believe that POCs such as 6FT‐RCC3 have strong promise for real‐life SO_2_ capture.

## Conflict of interest

The authors declare the following competing financial interest(s): A.I.C. and M.L. have a financial interest in the start‐up company CageCapture Ltd, which is seeking to commercialize porous organic cages.

## Supporting information

As a service to our authors and readers, this journal provides supporting information supplied by the authors. Such materials are peer reviewed and may be re‐organized for online delivery, but are not copy‐edited or typeset. Technical support issues arising from supporting information (other than missing files) should be addressed to the authors.

Supporting Information
