# Supplementary material for: SO2 Capture Using Porous Organic Cages
Source: Angew Chem Int Ed Engl. 2021 Jun 10;60(32):17556–63. doi: 10.1002/anie.202104555 (PMC8361948; doi:10.1002/anie.202104555)
Supplement: Supplementary file 1 — Supporting Information [file ANIE-60-17556-s001.pdf]

Angewandte Chemie
Eine Zeitschrift der Gesellschaft Deutscher Chemiker

Supporting Information

SO_2 Capture Using Porous Organic Cages

Eva Martínez-Ahumada[+], Donglin He[+], Victoria Berryman, Alfredo López-Olvera, Magali Hernandez, Vojtech Jancik, Vladimir Martis, Marco A. Vera, Enrique Lima, Douglas J. Parker, Andrew I. Cooper, Ilich A. Ibarra,* and Ming Liu**

anie_202104555_sm_miscellaneous_information.pdf

Supporting Information
Table of Contents

Experimental Procedures 1

Results and Discussion 3

Scheme S1. Chemical structures of CC3, RCC3 and 6FT-RCC3 cages. 3

Figure S1. Powder X-Ray diffraction patterns of CC3 (a), RCC3 (b), and 6FT-CC3 (c) cages. 4

Figure S2. SO_2 adsorption isotherms of CC3 (a), RCC3 (c), and 6FT-CC3 (e) cages at low pressure and 298 K and 308 K, respectively. Virial fitting plot of CC3 (b), RCC3 (d), and 6FT-CC3 (e) cages. 5

Figure S3. Comparison of SO_2 adsorptions against BET surface area of some reported porous materials at 298 K and 1 bar. 6

Figure S4. Comparison of SO_2 uptakes of some reported porous materials at 298 K and 0.1 bar. Blue column (C3); green column (RCC3); red column (6FT-RCC3). 6

Figure S5. Cycling static SO_2 adsorption of RCC3 (a) and 6FT-RCC3 (b) at 298 K and 1 bar, activated under dynamic vacuum without heating. 7

Figure S6. Fifty adsorption–desorption cycles for SO_2 in CC3 at 298 K. SO_2 was fully desorbed under dynamic vacuum and at 298 K between cycles, and no loss of uptake capacity was observed. 7

Figure S7. FT-IR spectra of fresh, SO_2-loaded and regenerated CC3 (a), RCC3 (b) and (c) 6FT-RCC3 porous organic cages in the 3400-400 cm^{-1} wavelength region. 9

Figure S8. ^{1}H NMR spectra for CC3 porous organic cage after 50 ads-des cycles in $CDCl_3$ at 400 MHz. 10

Figure S9. ^{13}C DEPTQ NMR spectra for CC3 porous organic cage after 50 SO_2 ads-des cycles in $CDCl_3$ at 100 MHz. 10

Figure S10. ^{1}H NMR spectra for 6FT-RCC3 porous organic cage after SO_2 adsorption, in $CDCl_3$ at 400 MHz.. 11

Figure S11. ^{13}C DEPTQ NMR spectra for 6FT-RCC3 porous organic cage after SO_2 adsorption in $CDCl_3$ at 100 MHz. 11

Figure S12. ^{13}C CP MAS NMR spectra of 6FT-RCC3 porous organic cage. * Indicates spinning side band (6 kHz). 12

Figure S13. N_2 adsorption isotherm at 77 K of 6FT-RCC3 porous organic cage after the 50 SO_2 ads-des cycles. Closed symbols (adsorption isotherm), open symbols (desorption isotherm). 12

Figure S14 N_2 adsorption isotherm of 6FT-RCC3 porous organic cage at 77 K after SO_2-H_2O exposure. Closed symbols (adsorption isotherm), open symbols (desorption isotherm). 13

Figure S15. Homemade system for wet SO_2 adsorption experiments. 13

Table S1. SO_2 adsorption capacity of some related MOFs at 1 bar. 14

References 14

Author contributions 15

Experimental Procedures

Materials

1,3,5-Triformylbenzene and (1*R*,2*R*)-Cyclohexane-1,2-diamine were purchased from Manchester Organics, UK. All other chemicals were purchased from Sigma-Aldrich and used as received.

Synthesis of CC3: CC3-R was prepared as previously reported in its homochiral form.[1] Dichloromethane (100 ml) was layered slowly onto solid triformylbenzene (TFB, 5 g, 30.86 mmol) without stirring at room temperature. Trifluoroacetic acid (1 mL) was added directly to this solution as a catalyst for the imine bond formation. Finally, a solution of (*R*,*R*)-1,2-diaminocyclohexane (5 g, 44.64 mmol) in dichloromethane (100 mL) was added to this, again without mixing. The reaction was covered and left to stand. Over 5 days, all of the

solid triformylbenzene was used up and octahedral crystals of CC3 grew on the sides of the glass reaction vessel. The crystalline product was removed by filtration and washed with 95 % ethanol / 5 % dichloromethane.

A large-scale synthesis of CC3 has also been recently developed, a typical procedure is described as follows. A batch reactor was charged with 4,000 mL of isopropyl alcohol (IPA) and pre-heated to 50 °C. 1,3,5-Triformylbenzene (400g, 2.46 mol) and solid (*R*,*R*)-1,2-Diaminocyclohexane (431g, 3.77 mol) were added to the pre-heated solvent under continuous stirring. After stirring for five minutes, trifluoroacetic acid (8 mL, 0.047 mol) was added to the reaction mixture. Once the addition of the acid was complete the reaction is slowly ramped up to 78 °C and held for 17 hours with continuous stirring. After 17 hours the reactor is cooled to room temperature and the resulting off-white suspension is collected by vacuum filtration. This solid is then washed with further IPA and dried in a vacuum oven at 60 °C overnight to yield CC3 as a fine white powder. Yield 623 g, 94.7%.

Note: both synthesis methods give CC3 in the same polymorph, *alpha*-phase; hence both products exhibit the same porosity.

Synthesis of RCC3: RCC3 was prepared and purified as previously reported.[2] The imine cage CC3-*R* (926 mg, 0.83 mmol) was dissolved in a $CHCl_3$ / methanol mixture (1:1 v/v, 50 mL) by stirring. When this solution became clear, sodium borohydride (1.00 g, 26.5 mol) was added and the reaction was stirred for a further 12 hours at room temperature. Water (2 mL) was then added, and the reaction stirred for a further 12 hours. The solvent was then removed under vacuum. The resulting white solid was extracted with chloroform (2 × 50 mL) and then the combined organic phase was washed by water (2 × 100 mL). The $CHCl_3$ phase was dried using anhydrous $MgSO_4$ before being removed under vacuum. RCC3 (crude yield = 900 mg, 95.1 %) was obtained as a white solid. RCC3 was purified by using the reversible reaction with acetone. In a 250 mL flask, 1000 mg crude RCC3 was dissolved in acetone (100 mL). The solution was covered and left to stand. Crystals started appearing on the wall of the flask after 30 mins. The crystals (AT-RCC3) were collected after one day by filtration and were then dissolved in a $CHCl_3$ /CH_3OH mixture (1:1 v/v) by stirring. Several drops of distilled water were added to the solution and the mixture was stirred for another 12 h. After removal of the solvents, pure RCC3 (680 mg, 70.4 %) was recovered.

Synthesis of FT-RCC3. FT-RCC3 was prepared as previously reported.[2] Paraformaldehyde (52 mg, 20 eq.) dissolved in CH_3OH (10 mL) was stirred at 70 °C. To this clear solution was added RCC3 (100 mg) dissolved in CH_3OH (10 mL). A white precipitate appeared upon addition of RCC3. The reaction was stirred for a further 2 h at 70 °C. The reaction was cooled to room temperature and the precipitate was collected by filtration. FT-RCC3 (52 mg, 70 %) was obtained after being washed with CH_3OH (3 × 10 mL) and dried under vacuum.

SO_2 adsorption isotherms were measured in a Dynamic Gravimetric Gas/Vapour Sorption Analyser, DVS vacuum (Surface Measurement Systems Ltd) with static method. The samples were activated at 80 °C under vacuum ($1 \cdot 10^{-6}$ bar) for 4 hours. SO_2 adsorption isotherms were carried out at 298 K up to 1 bar. Main Text Paragraph.

Heat of Adsorption. Additional 308 K SO_2 adsorption isotherms were measured to estimate the heat of adsorption on all the samples (Fig. S2), a virial-type equation was used to fit the adsorption isotherms at low surface coverage, to estimate the heat of adsorption at zero coverage (Fig. S3).

N_2 adsorption isotherms were recorded at 77 K up to P/P_0 = 1, on a Quantachrome Autosorb MP-1 equipment under high vacuum in a clean system with a diaphragm pumping system.

Powder X-ray diffraction (PXRD) patterns were measured on a Bruker D8 Advance X-ray diffractometer equipped with a LynxEye detector using CuKα radiation (λ = 1.5406 Å; monochromator: germanium) in a range 2-theta of 4−60° with a step of 0.02°. The voltage and current were 35 kV and 35 mA, respectively.

Fourier transform infrared (FTIR) spectroscopy was carried out using a Nicolet 6700 spectrometer. The spectrum was generated and collected 16 times and corrected for the background noise in the wavenumber ranging from 400 to 3400 cm^{-1}.

The ^{13}C CP/MAS NMR spectra were acquired at a frequency of 75.422 MHz at a spinning rate of 6 kHz. Typical ^{13}C CP/MAS NMR conditions for ^{1}H–^{13}C polarization experiment used a π/2 pulse of 4 μs, contact time of 1 ms, delay time of 5 s, and at least 20 000 scans. Chemical shifts were referenced to a solid shift at 38.2 ppm relative to TMS.

Solution NMR spectra were recorder using a Bruker advance 400 NMR Ultrashield^{TM} spectrometer, at 400 MHz for ^{1}H and 100 MHz for ^{13}C-DEPTQ.

Theoretical calculations were carried out using density functional theory methods and employing the Gaussian 16 software package.[3] Calculations used the PBE[4] density functional approximation with Ahlrich's def2-TZVP basis set of a polarized triple-ζ quality.[5]

Results and Discussion

Scheme S1. Chemical structures of CC3, RCC3 and 6FT-RCC3 cages.

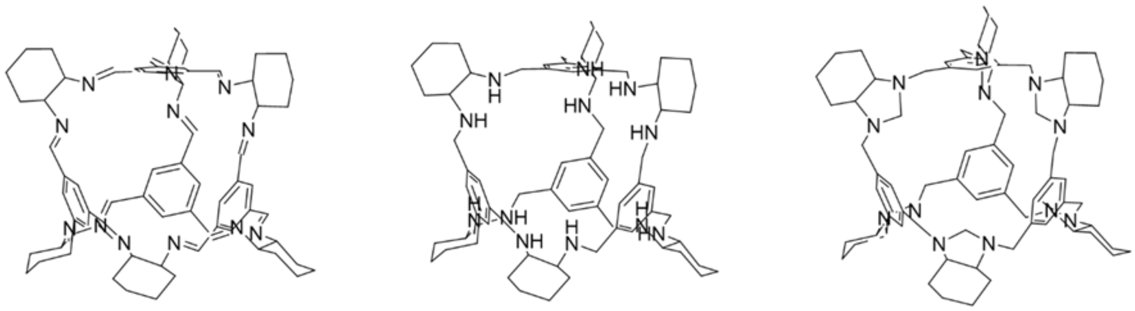

Chemical Formula: $C_{72}H_{84}N_{12}$
Molecular Weight: 1117.55
CC3

Chemical Formula: $C_{72}H_{108}N_{12}$
Molecular Weight: 1141.74
RCC3

Chemical Formula: $C_{78}H_{108}N_{12}$
Molecular Weight: 1213.81
6FT-RCC3

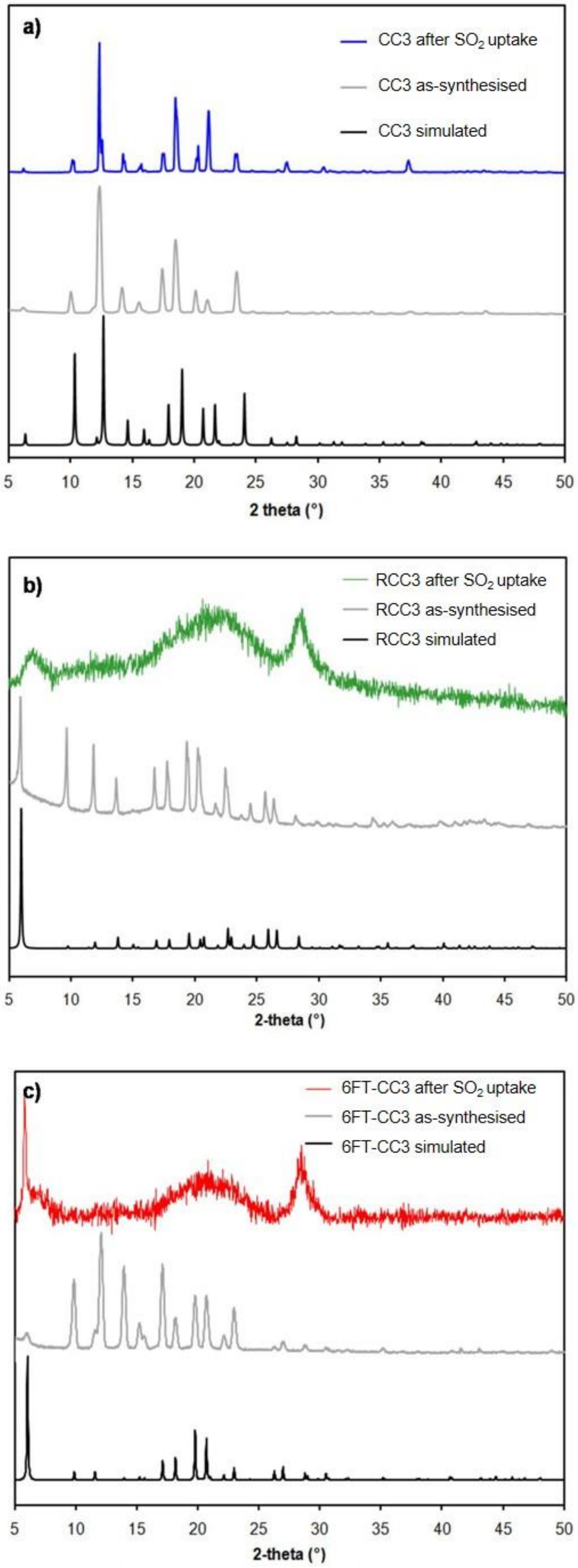

Figure S1. Powder X-Ray diffraction patterns of CC3 (a), RCC3 (b), and 6FT-RCC3 (c) cages after SO_2 adsorption.

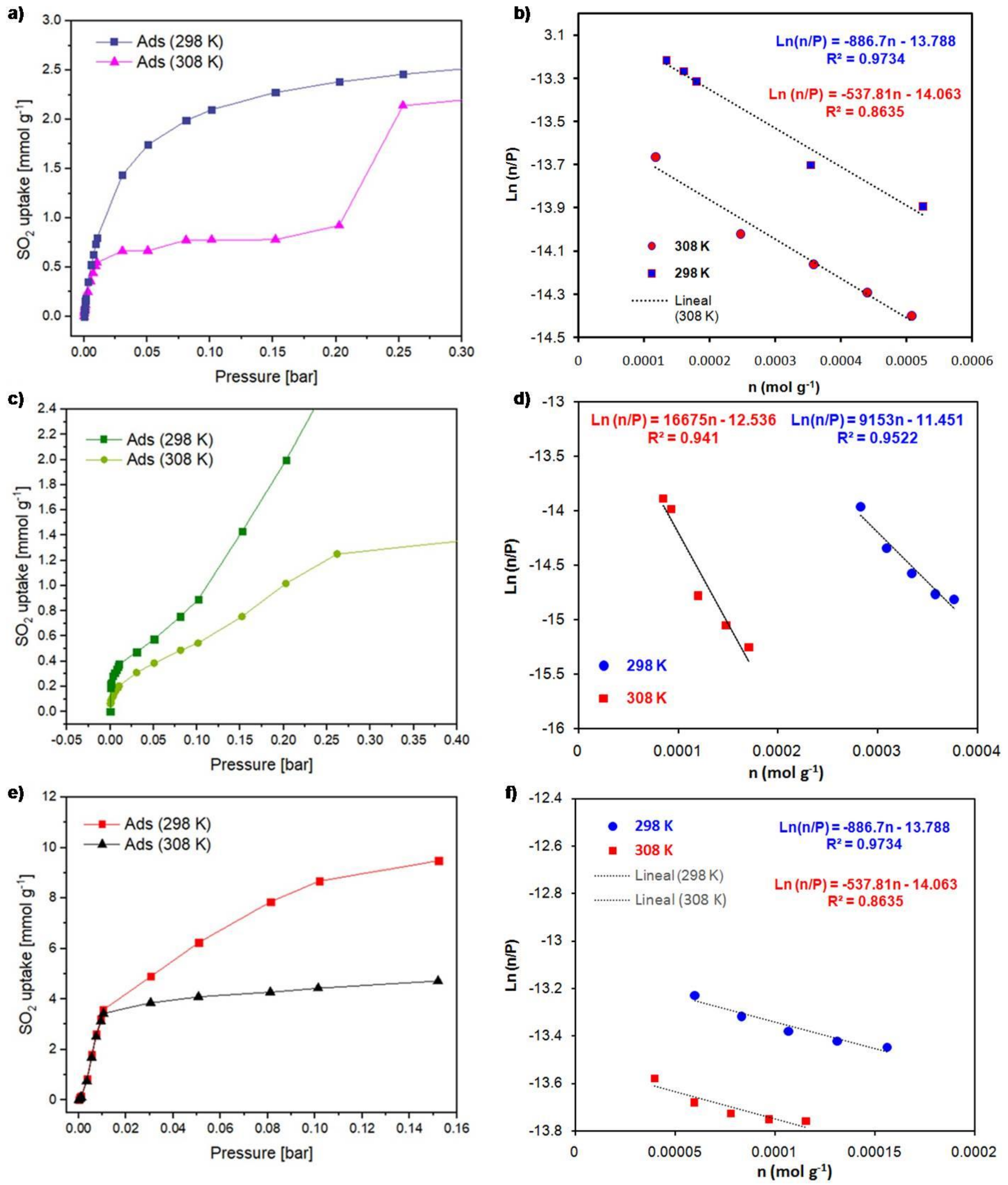

Figure S2. SO_2 adsorption isotherms of CC3 (a), RCC3 (c), and 6FT-CC3 (e) cages at low pressure and 298 K and 308 K, respectively. Virial fitting plot of CC3 (b), RCC3 (d), and 6FT-CC3 (e) cages.

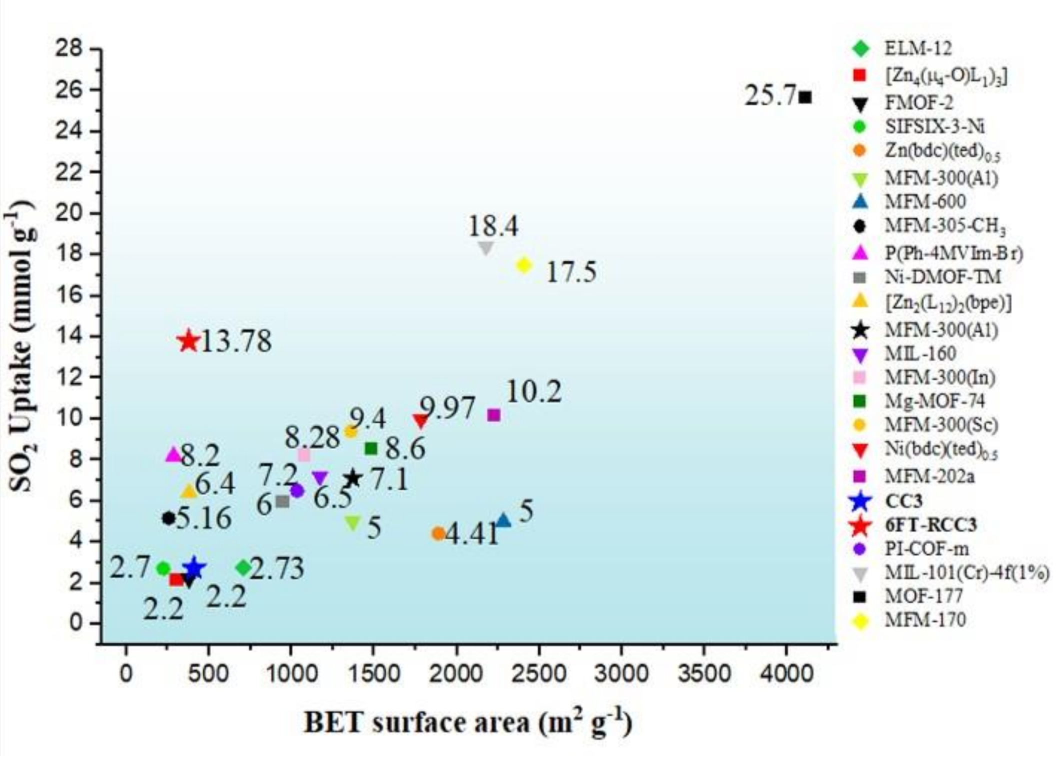

Figure S3. Comparison of SO_2 adsorptions against BET surface area (m^2 g^{-1}) of some reported porous materials at 298 K and 1 bar. 6FT-RCC3 is denoted by the red star.

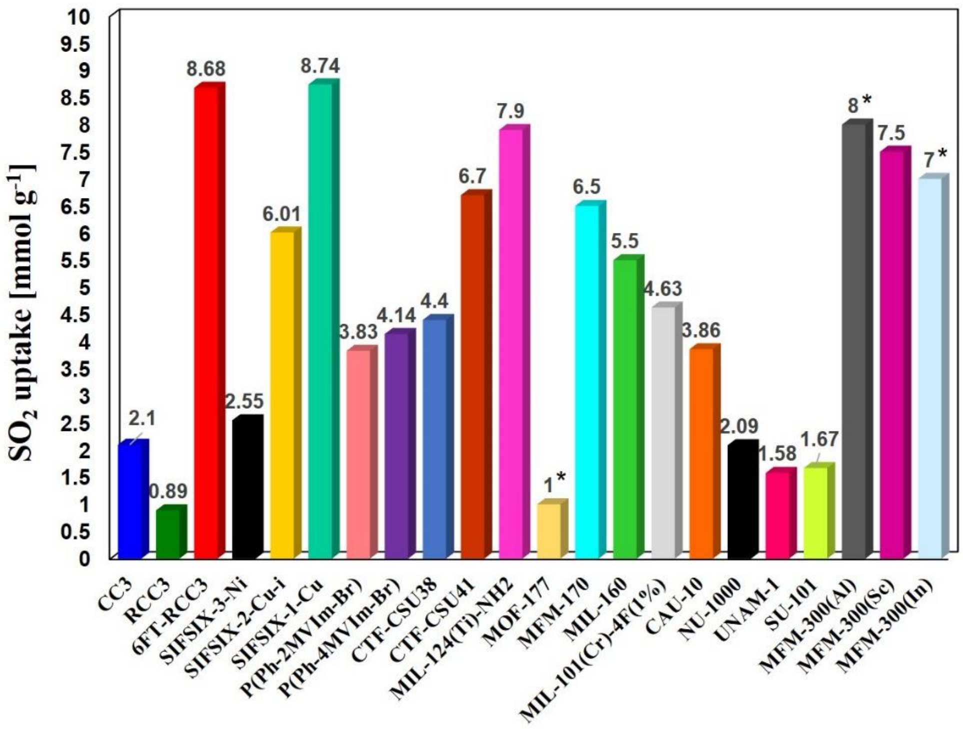

Figure S4. Comparison of SO_2 uptakes of some reported porous materials at 298 K and 0.1 bar. Blue column (C3); green column (RCC3); red column (6FT-RCC3). FT-RCC3 is particularly competitive at lower partial pressure of SO_2. *Estimated from isotherm.

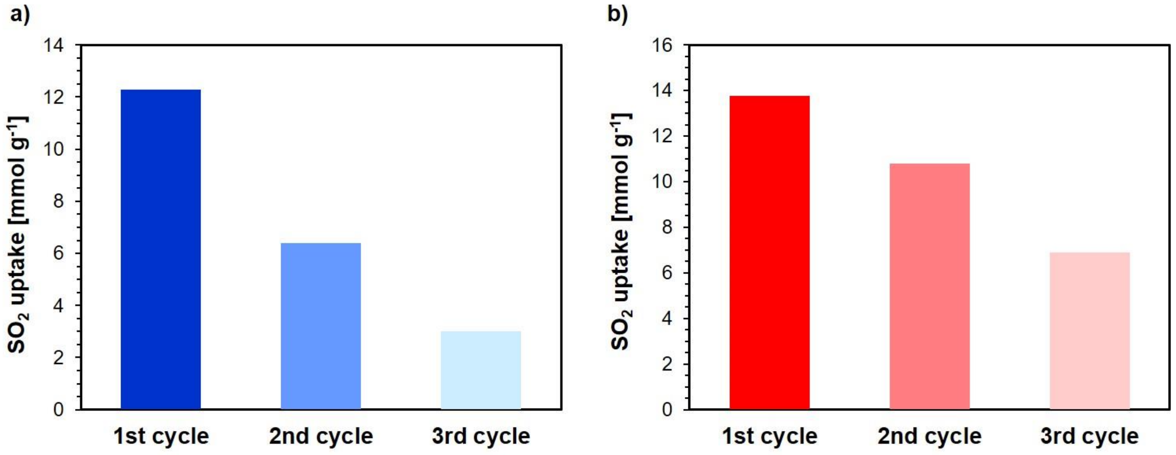

Figure S5. Cycling static SO_2 adsorption of RCC3 (a) and 6FT-RCC3 (b) at 298 K and 1 bar, activated under dynamic vacuum without heating.

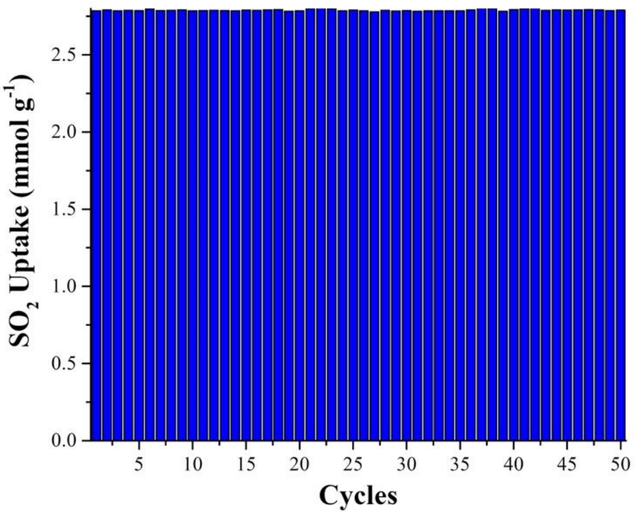

Figure S6. Fifty adsorption–desorption cycles for SO_2 in CC3 at 298 K. SO_2 was fully desorbed under dynamic vacuum and at 298 K between cycles; no loss of uptake capacity was observed.

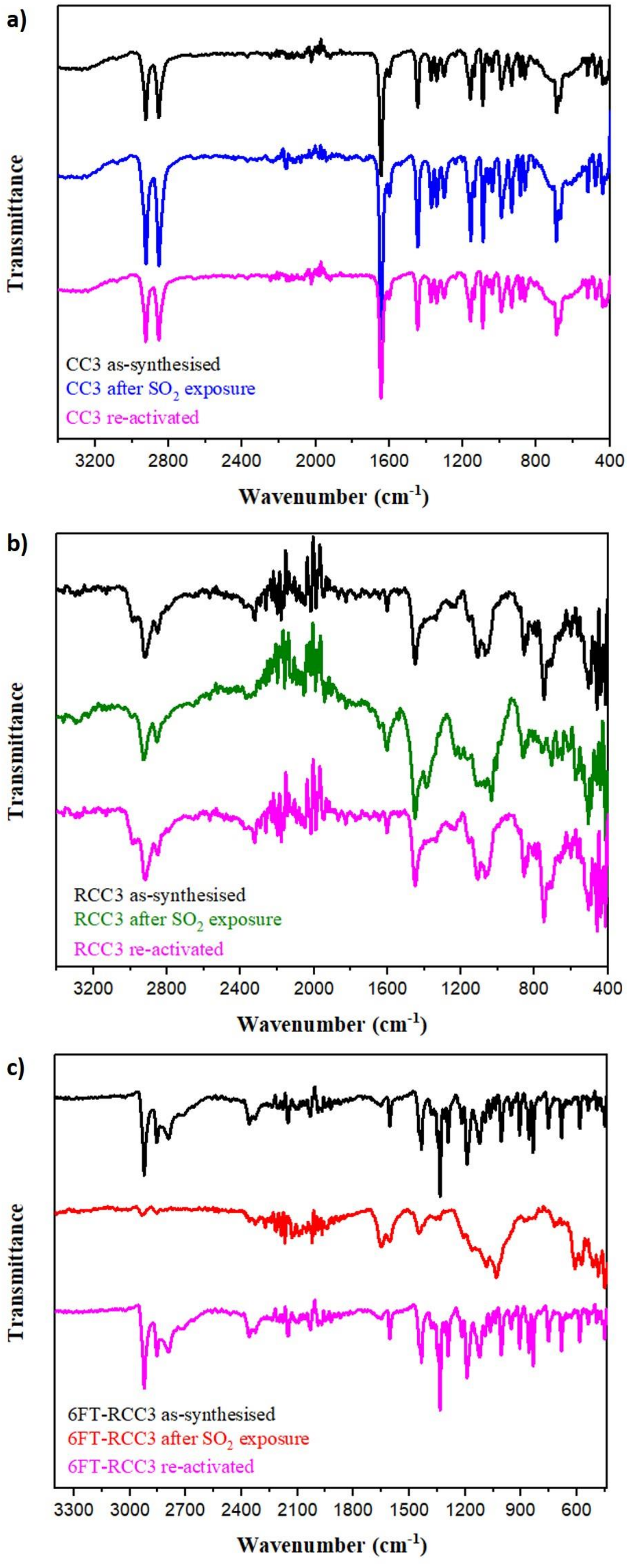

Figure S7. FT-IR spectra of fresh, SO_2-loaded and regenerated CC3 (a), RCC3 (b) and (c) 6FT-RCC3 porous organic cages in the 3400-400 cm^{-1} wavelength region.

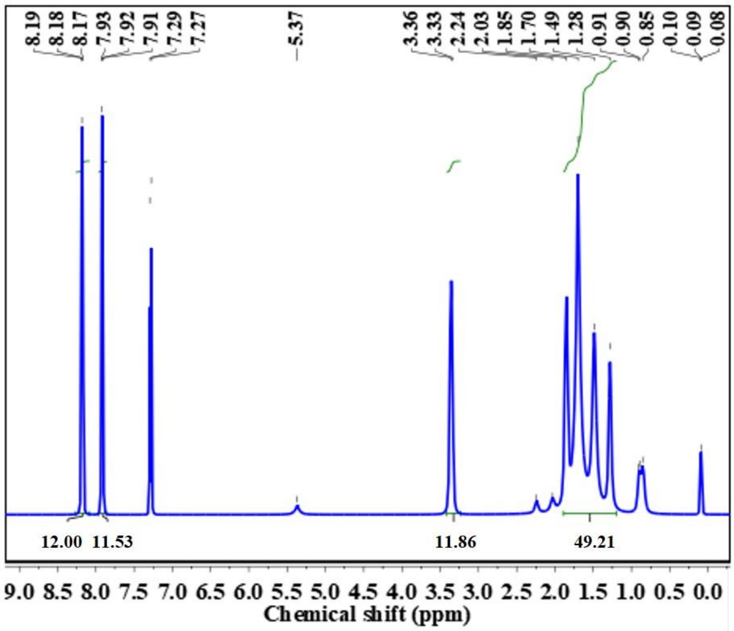

Fig. S8. ^{1}H NMR spectra for CC3 porous organic cage after SO_2 adsorption, in $CDCl_3$ at 400 MHz.

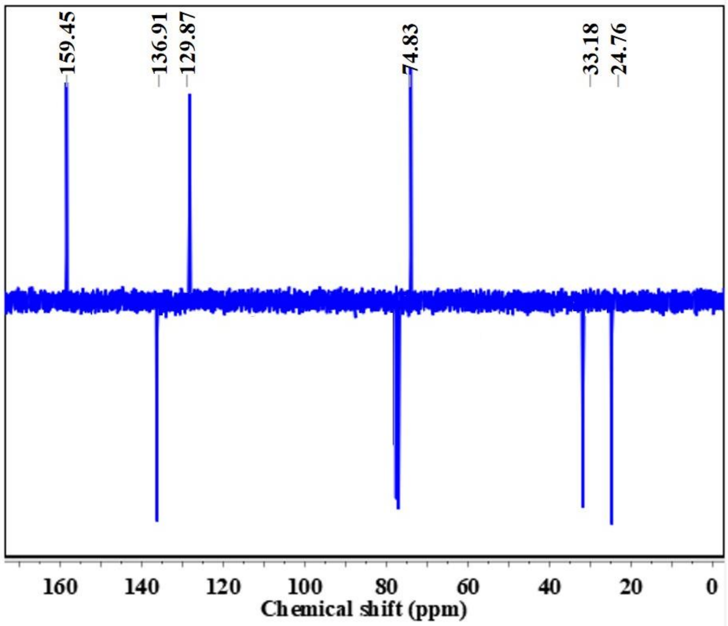

Fig. S9. ^{13}C DEPTQ NMR spectra for CC3 porous organic cage after SO_2 adsorption in $CDCl_3$ at 100 MHz.

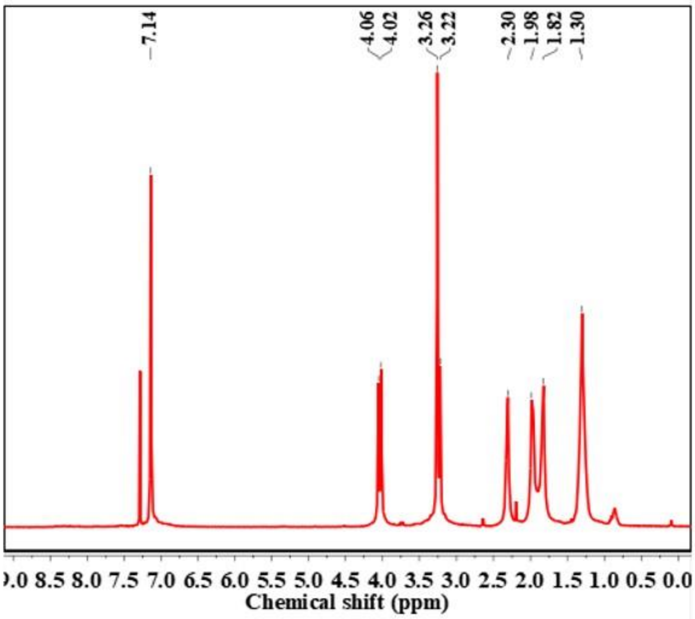

Fig. S10. ^{1}H NMR spectra for 6FT-RCC3 porous organic cage after 50 ads-des cycles in $CDCl_3$ at 400 MHz.

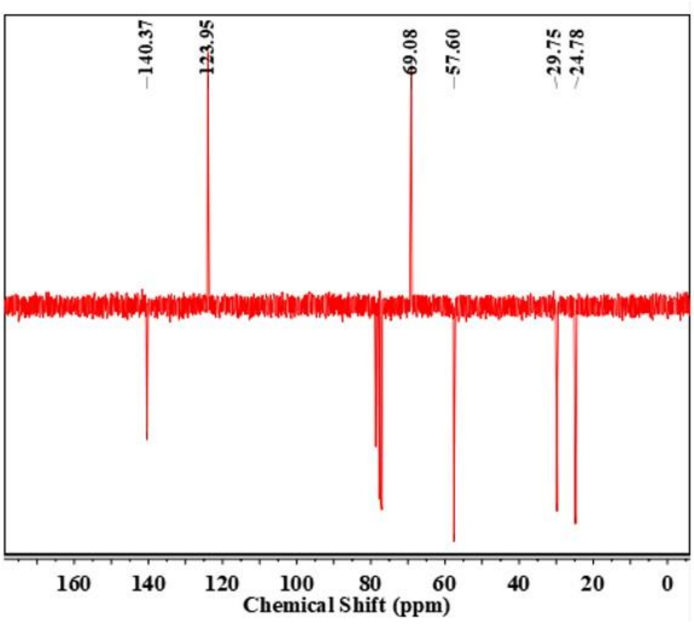

Fig. S11. ^{13}C DEPTQ NMR spectra for 6FT-RCC3 porous organic cage after 50 ads-des cycles in $CDCl_3$ at 100 MHz.

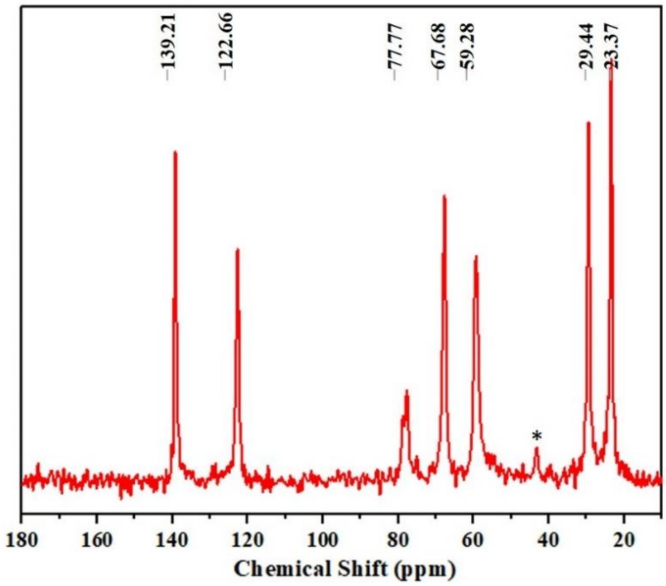

Fig. S12. ^{13}C CP MAS NMR spectra of 6FT-RCC3 porous organic cage after SO_2 adsorption and activation. * Indicates spinning side band (6 kHz).

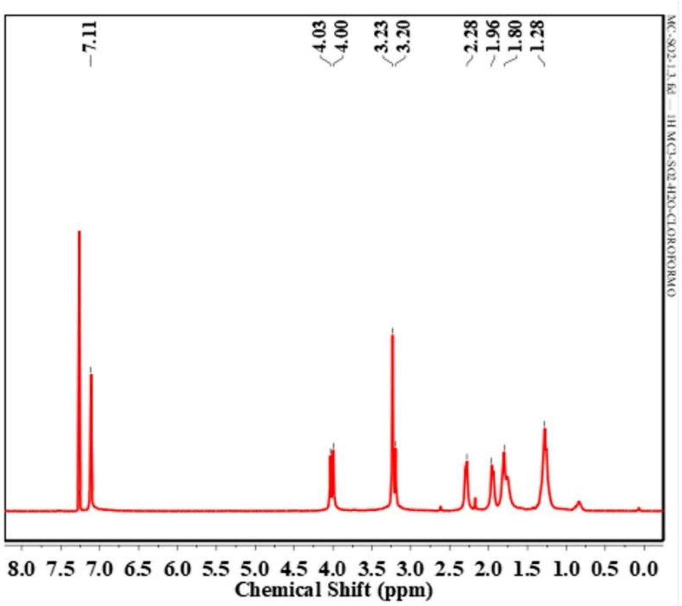

Fig. S13. ^{1}H NMR spectra for 6FT-RCC3 porous organic cage after SO_2 and H_2O vapor exposure in $CDCl_3$ at 400 MHz.

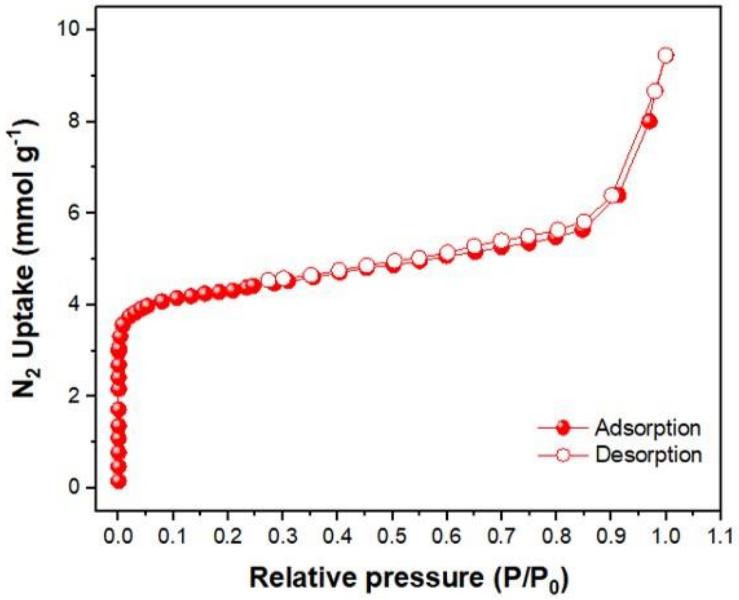

Fig. S14. N_2 adsorption isotherm at 77 K of 6FT-RCC3 porous organic cage after the 50 ads-des cycles. Closed symbols (adsorption isotherm), open symbols (desorption isotherm).

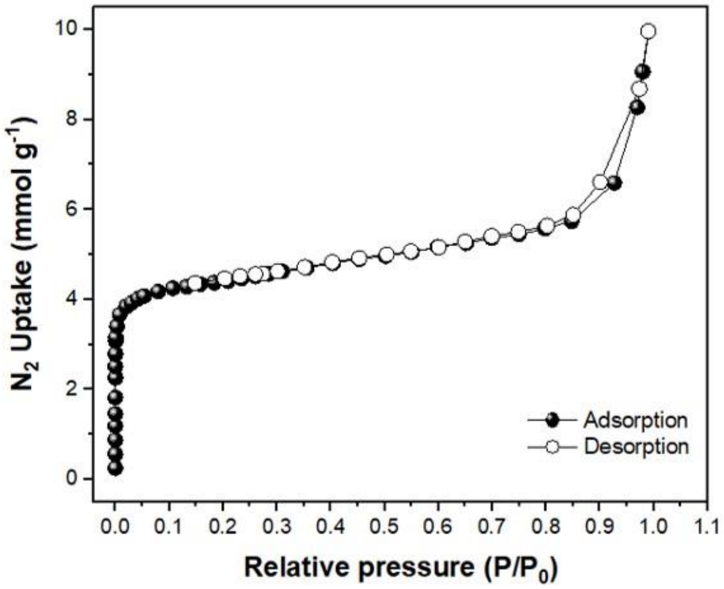

Fig. S15. N_2 adsorption isotherm of 6FT-RCC3 porous organic cage at 77 K after SO_2-H_2O exposure. Closed symbols (adsorption isotherm), open symbols (desorption isotherm).

Additional experiments

In order to evaluate the stability of 6FT-RCC3 cage in the presence of SO_2 and humidity, we carried out a qualitative experiment with a homemade system previously reported (see Fig. S16).[6], [7] The system contains two principal parts: SO_2 gas generator (A) dropping funnel with H_2O. [1] connected to a Schlenk flask with Na_2SO_3(s) under stirring [2]; and the saturation chamber (B), constructed from a round flask with distilled water [3], connected to a sintered glass filter adapter (without humidity trap) [4] and to a vacuum line [5]. The activated cage sample is placed on the glass filter adapter and exposed for 3 days to SO_2 and H_2O vapor. After, N_2 adsorption was evaluated (see Fig. S15). In comparison to the reported for 6F-RCC3 cage,[2] the BET surface area decreased from 396 to 375 m^2 g^{-1} after the exposure to SO_2 and H_2O. No apparent changes were observed in porous organic cage after exposure to SO_2 and water stays intact as confirmed by ^{1}H NMR (Fig. S13).

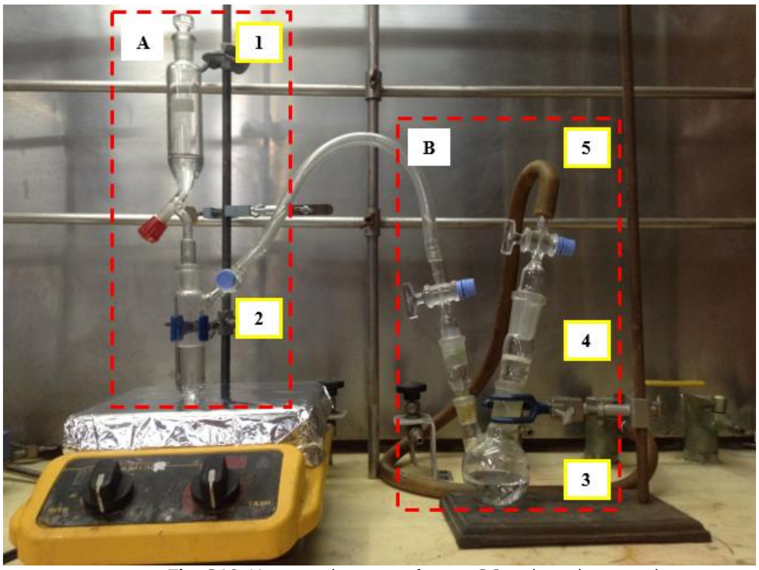

Fig. S16. Homemade system for wet SO_2 adsorption experiments.

Table S1. SO_2 adsorption capacity of some related MOFs at 1 bar.

Material	BET surface area [m^2 g^{-1}]	SO_2 uptake [mmol g^{-1}]	Temperature [K]	Reference
ELM-12	706	2.73	298	8
[Zn_4(μ_4-O)(L_1)$_3$]	299[a]	2.2	293	9
SU-101	350	2.2	298	6
FMOF-2	378	2.2	298	10
SIFSIX-3-Ni	223	2.7	298	11
CC3	402	2.78	298	This work
Zn(bdc)(ted)$_{0.5}$	1888	4.41	298	12
CAU-10	625	4.47	298	13
MFM-300(Al)	1370	5	298	14
MIL-160	1170	7.2	293	15
MFM-300(In)	1071	8.28	298	16
Mg-MOF-74	1475	8.6	298	12
MFM-300(Sc)	1360	9.4	298	17
Ni(bdc)(ted)$_{0.5}$	1783	9.97	298	12
MFM-202a	2220	10.2	298	18
MFM-601	3644	12.3	298	19
ECUT	760.3	8.0	298	20
RCC3	---	12.34	298	This work
6FT-RCC3	396	13.78	298	This work

[a] Langmuir surface area

References

[1] J. T. A. Jones, T. Hasell, X. Wu, J. Bacsa, K. E. Jelfs, M. Schmidtmann, S. Y. Chong, D. J. Adams, A. Trewin, F. Schiffman, F. Cora, B. Slater, A. Steiner, G. M. Day and A. I. Cooper, *Nature,* **2011**, *474*, 367-371.
[2] M. Liu, M. A. Little, K. E. Jelfs, J. T. A. Jones, M. Schmidtmann, S. Y. Chong, T. Hasell and A. I. Cooper, *J. Am. Chem. Soc.,* **2014**, *136,* 7583-7586.
[3] Gaussian 16, Revision A.03 M. J. Frisch, G. W. Trucks, H. B. Schlegel, G. E. Scuseria, M. A. Robb, J. R. Cheeseman, G. Scalmani, V. Barone, G. A. Petersson, H. Nakatsuji, X. Li, M. Caricato, A. V. Marenich, J. Bloino, B. G. Janesko, R. Gomperts, B. Mennucci, H. P. Hratchian, J. V. Ortiz, A. F. Izmaylov, J. L. Sonnenberg, D. Williams-Young, F. Ding, F. Lipparini, F. Egidi, J. Goings, B. Peng, A. Petrone, T. Henderson, D. Ranasinghe, V. G. Zakrzewski, J. Gao, N. Rega, G. Zheng, W. Liang, M. Hada, M. Ehara, K. Toyota, R. Fukuda, J. Hasegawa, M. Ishida, T. Nakajima, Y. Honda, O. Kitao, H. Nakai, T. Vreven, K. Throssell, J. A. Montgomery, Jr., J. E. Peralta, F. Ogliaro, M. J. Bearpark, J. J. Heyd, E. N. Brothers, K. N. Kudin, V. N. Staroverov, T. A. Keith, R. Kobayashi, J. Normand, K. Raghavachari, A. P. Rendell, J. C. Burant, S. S. Iyengar, J. Tomasi, M. Cossi, J. M. Millam, M. Klene, C. Adamo, R. Cammi, J. W. Ochterski, R. L. Martin, K. Morokuma, O. Farkas, J. B. Foresman, and D. J. Fox, Gaussian, Inc., Wallingford CT**, 2016**.
[4] a) J. P. Perdew, K. Burke, and M. Ernzerhof, Phys. Rev. Lett., 1996, 77, 3865-3868. b) J. P. Perdew, K. Burke, and M. Ernzerhof, Phys. Rev. Lett., 1997, 78, 1396.
[5] a) F. Weigend and R. Ahlrichs, Phys. Chem. Chem. Phys., 2005, 7, 3297-3305. b) F. Weigend, Phys. Chem. Chem. Phys., 2006, 8, 1057-1065.
[6] E. S. Grape, J. G. Flores, T. Hidalgo, E. Martínez-Ahumada, A. Gutiérrez-Alejandre, A. Hautier, D. R. Williams, M. O´Keeffe, L. Öhrström, T. Willhammar, P. Horcajada, I. A. Ibarra, and A. K. Inge *J. Am. Chem. Soc*. **2020**, *142*, 16795−16804.
[7] E. Martínez-Ahumada, M. L. Díaz-Ramírez, H. A. Lara-García, D. R. Williams, V. Martis, V. Jancik, E. Lima and I. A. Ibarra, *J. Mater. Chem. A*, **2020**, *8*, 11515-11520.
[8] Y. Zhang, P. Zhang, W. Yu, J. Zhang, J. Huang, J. Wang, M. Xu, Q. Deng, Z. Zeng, and S. Deng. *ACS Appl. Mater. Interfaces* **2019**, *11*, 10680–10688.
[9] S. Glomb, D. Woschko, G. Makhloufi, and C. Janiak. *ACS Appl. Mater. Interfaces* **2017**, *9*, 37419–37434.
[10] C. A. Fernandez, P. K. Thallapally, R. K. Motkuri, S. K. Nune, J. C. Sumrak, J. Tian, and J. Liu. *Cryst. Growth Des*. **2010**, *10*, 1037–1039.
[11] X. Cui, Q. Yang, L. Yang, R. Krishna, Z. Zhang, Z. Bao, H. Wu, Q. Ren, W. Zhou, B. Chen, and H. Xing. *Adv. Mater*. **2017**, *29*, 1606929.
[12] K. Tan, P. Canepa, Q. Gong, J. Liu, D. H. Johonson, A. Dyevoich, P. K. Thallapally, T. Thonhauser, J. Li and Y. J. Chabal. *Chem. Mater*. **2013**, *25*, 4653−4662.

[13] J. A. Zárate, E. Domínguez-Ojeda, E. Sánchez-González, E. Martínez-Ahumada, V. B. López-Cervantes, D. R. Williams, V. Martis, I. A. Ibarra, and J. Alejandre. *Dalton Trans.,* **2020,** *49*, 9203-9207.

[14] S. Yang, J. Sun, A. J. Ramírez-Cuesta, S. K. Callear, W. I. F. D. P. Anderson, R. Newby, A. J. Blake, J. E. Parker, C. C. Tang, and M. Schroder. *Nat. Chem*. **2012**, *4*, 887–894.

[15] P. Brandt, A. Nuhnen, M. Lange, J. Mollmer, O. Weingart, and C. Janiak. *ACS Appl. Mater. Interfaces* **2019**, *11*, 17350–17358.

[16] M. Savage, Y. Cheng, T. L. Easun, J. E. Eyley, S. P. Argent, M. R. Warren, W. Lewis, C. Murray, C. C. Tang, M. D. Flogley, G. Cinque, J. Sun, S. Rudic, R. T. Murden, M. J. Benham, A. N. Fitch, A. J. Blake, A. J. Ramírez-Cuesta, S. Yang, and M. Schröder. *Adv. Mater*. **2016**, *28*, 8705–8711.

[17] J. A. Zárate, E. Sánchez-González, D. R. Williams, E. González-Zamora, V. Martis, A. Martínez, J. Balmaseda, G. Maurin, and I. A. Ibarra. *J. Mater. Chem. A*, **2019**, *7*, 15580-15584.

[18] S. Yang, L. Liu, J. Sun, K. M. Thomas, A. J. Davies, M. W. George, A. George, A. J. Blake, A. H. Hill, A. N. Fitch, C. C. Tang, and M. Schröder. *J. Am. Chem. Soc***. 2013**, *135*, 4954–4957.

[19] J.H. Carter, X. Han, F.Y. Moreau, I. da Silva, A. Nevin, H.G.W. Godfrey, C.C. Tang, S. Yang, M. Schröder*, J. Am. Chem. Soc*. **2018**, 140, 15564–15567.

[20] Y. L. Fan, H. P. Zhang, M. J. Yin, R. Krishna, X. F. Feng, L. Wang, M. B. Luo, and F. Luo*, Inorg. Chem*. **2021,** *60*, 4–8.

Author Contributions

Andrew I. Cooper, Ilich A. Ibarra, and Ming Liu carried out the project administration, investigation, validation and writing of original draft in equal contribution (lead). Donglin He, Douglas J. Parker, Ming Liu carried out the synthesis of materials, data curation, and formal analysis. Victoria Berryman carried out the computational analysis, data curation, and formal analysis. Marco A. Vera and Enrique Lima carried out the NMR experiments, data curation, and formal analysis. Eva Martínez-Ahumada and Vladimir Martis: carried out the sulfur dioxide adsorption studies and FTIR experiments, data curation, and formal analysis. Vojtech Jancik carried out the PXRD analysis, validation and writing of original draft (supporting). Eva Martínez-Ahumada and Donglin He also contributed to the investigation and the writing of original draft.
